# Association Between β1‐Selective Beta‐Blocker Use and P‐Wave Terminal Force in Lead V1 in Hypertensive Patients With Left Ventricular Hypertrophy

**DOI:** 10.1155/ijhy/2095472

**Published:** 2026-10-26

**Authors:** Erkan Alpaslan, Sedat Taş, Ümmü Taş, Mehmet Eyüboğlu, Ömer Özkan Duman, Onur Dalgıç, Lütfullah Candan, Ahmet Rıdvan Özdemir

**Affiliations:** ^1^ Department of Cardiology Faculty of Medicine İzmir Demokrasi University İzmir Türkiye; ^2^ Department of Cardiology Buca Seyfi Demirsoy Training and Research Hospital İzmir Türkiye

**Keywords:** *β*1-selective beta-blocker, atrial remodeling, electrocardiography, hypertension, left ventricular hypertrophy, PTFV1

## Abstract

**Background:**

P‐wave terminal force in lead V1 (PTFV1) is an electrocardiographic marker of atrial electrical abnormality. We investigated whether *β*1‐selective beta‐blocker use was associated with PTFV1 in hypertensive patients with echocardiographically confirmed left ventricular hypertrophy (LVH).

**Methods:**

This retrospective study included 182 eligible patients with essential hypertension and LVH selected from 1372 screened patients. Patients were classified as *β*1‐selective beta‐blocker users (*n* = 82) or nonusers (*n* = 100). Abnormal PTFV1 was defined as ≥ 0.04 mm s. Multivariable regression and sensitivity analyses were performed.

**Results:**

PTFV1 was lower in beta‐blocker users than in nonusers (0.024 [0.015–0.036] vs. 0.030 [0.020–0.048] mm s, *p* = 0.005). In the primary adjusted model, beta‐blocker use was associated with lower PTFV1 (*B* = −0.008, standardized *β* = −0.202, 95% CI −0.014 to −0.002, *p* = 0.009), although model explanatory capacity was limited (*R* ^2^ = 0.064; adjusted *R* ^2^ = 0.021; overall *p* = 0.168). In an extended sensitivity analysis additionally adjusting for diabetes, renal function, and concomitant antihypertensive therapies, beta‐blocker group status remained associated with lnPTFV1 (standardized *β* = 0.294, *p* < 0.001; *R* ^2^ = 0.147; adjusted *R* ^2^ = 0.081; overall *p* = 0.011). Beta‐blocker use was also associated with lower odds of abnormal PTFV1 (OR = 0.43, 95% CI 0.22–0.84, *p* = 0.013).

**Conclusion:**

*β*1‐Selective beta‐blocker use was associated with lower PTFV1 and a lower likelihood of abnormal PTFV1. These findings suggest a more favorable atrial electrical profile but should be considered hypothesis‐generating given the retrospective design and modest model explanatory capacity.

## 1. Introduction

Hypertension is one of the major contributors to new‐onset atrial fibrillation (NOAF), with reported incidence rates ranging from 6% to 21% [1]. The coexistence of hypertension and AF is associated with worse short‐ and long‐term clinical outcomes [2, 3]. Owing to its high prevalence and frequent coexistence with AF in the general population, hypertension accounts for a substantial proportion of AF cases. However, the development of AF is multifactorial, and its underlying mechanisms are not fully understood [4, 5]. Therefore, early identification of hypertensive patients at increased risk of atrial remodeling and subsequent AF remains clinically important. In the LIFE study, the incidence of AF was approximately 7.8% among hypertensive patients with left ventricular hypertrophy (LVH), and patients who subsequently developed AF or heart failure were shown to have particularly high cardiovascular risk [6].

Atrial dysfunction and remodeling play an important role in mediating adverse cardiovascular outcomes in hypertensive patients with LVH. In line with these observations, Nattel et al. emphasized that atrial remodeling is a key substrate for atrial fibrillation and related cardiac events [7]. Bang et al. reported that reduced left atrial systolic force in hypertensive patients with LVH identified a subgroup with advanced left atrial dysfunction and an increased risk of NOAF [8]. Similarly, Soullier et al. demonstrated that hypertensive patients with LVH had impairment in all three components of left atrial function, including reservoir, conduit, and booster pump functions. These changes were associated with LVH and left ventricular dysfunction, suggesting an important role of left atrial–left ventricular coupling and possible atrial fibrotic alterations [9]. These findings highlight the importance of early assessment of atrial structural and functional changes in hypertensive patients with LVH.

P‐wave terminal force in lead V1 (PTFV1) is an electrocardiographic measure derived from the duration and amplitude of the terminal negative P‐wave component in lead V1. Abnormal PTFV1 has been associated with left atrial structural and functional measures in previous studies [10]. However, PTFV1 does not directly measure atrial size, function, or fibrosis. Because it can be obtained from a standard 12‐lead electrocardiogram, it is a readily accessible measure of atrial electrical abnormality. Previous studies have also reported associations between PTFV1 and cardiovascular outcomes, including atrial fibrillation and stroke [11, 12]. Prolonged P‐wave duration, a related but distinct electrocardiographic measure, has likewise been associated with atrial fibrillation and adverse cardiovascular outcomes [13, 14]. In the present study, PTFV1 was evaluated as an electrocardiographic measure rather than as evidence of structural or functional atrial remodeling.

Beta‐blockers may influence atrial electrophysiology through heart‐rate reduction, sympathetic modulation, and improvement in atrial conduction properties. Camsari et al. reported that long‐term metoprolol therapy was associated with reductions in maximum P‐wave duration and P‐wave dispersion in patients with congestive heart failure, suggesting a potential favorable effect on electrocardiographic markers of atrial conduction [15]. However, data regarding the relationship between β1‐selective beta‐blocker therapy and PTFV1 in hypertensive patients with LVH remain limited. Therefore, the present study aimed to investigate whether β1‐selective beta‐blocker use is associated with PTFV1 values and abnormal PTFV1 in hypertensive patients with echocardiographically confirmed LVH.

## 2. Methods

### 2.1. Study Design and Population

In this retrospective observational study, adult patients with essential hypertension who attended the cardiology outpatient clinic between October 2024 and October 2025 were screened. A total of 1372 hypertensive patients were identified during the study period. Of these, 845 patients had complete clinical, demographic, medication, electrocardiographic, and echocardiographic data. After applying the predefined eligibility criteria, 182 patients with echocardiographically confirmed LVH were included in the final analysis. The included patients were divided into two groups according to β1‐selective beta‐blocker use: 82 patients in the β1‐selective beta‐blocker group and 100 patients in the non–beta‐blocker group. The study was designed to investigate the association between β1‐selective beta‐blocker therapy and PTFV1 in hypertensive patients with LVH. The patient selection process is summarized in Figure 1.

**FIGURE 1:**
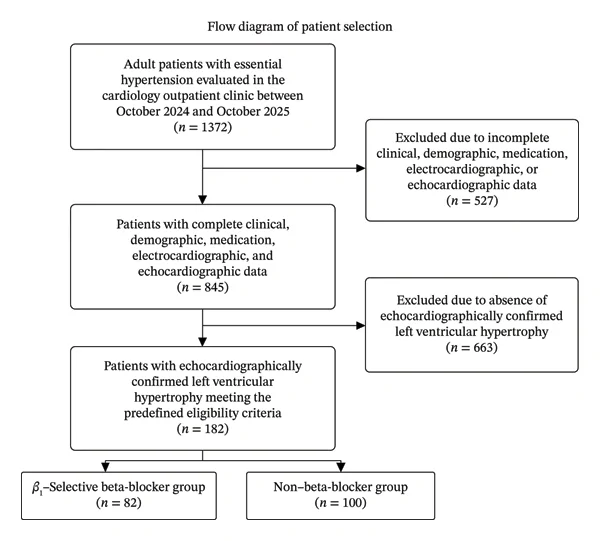
Flow diagram of patient selection illustrating the selection of patients with essential hypertension, including assessment of data completeness, exclusion of patients without echocardiographically confirmed left ventricular hypertrophy, and classification according to *β*1‐selective beta‐blocker use.

Patients were eligible for inclusion if they were older than 18 years, had a diagnosis of essential hypertension, had echocardiographic evidence of LVH, and had an available standard 12‐lead electrocardiogram suitable for PTFV1 measurement. Patients receiving β1‐selective beta‐blockers were required to have been on stable beta‐blocker therapy for at least 3 months before the index electrocardiogram. The index ECG was obtained at a follow‐up visit after this minimum treatment period, and transthoracic echocardiography performed during the same clinical visit was used for the study analysis. Patients in whom beta‐blocker therapy had been initiated within the preceding 3 months or at the index visit were excluded. Patients whose electrocardiographic or echocardiographic examinations did not meet the predefined study eligibility criteria were also excluded. The beta‐blocker group consisted of patients receiving metoprolol, bisoprolol, or nebivolol in addition to background antihypertensive therapy, including angiotensin‐converting enzyme inhibitors or angiotensin receptor blockers. Patients not receiving beta‐blockers constituted the comparison group.

Patients were excluded if they had atrial fibrillation or atrial flutter, moderate‐to‐severe valvular heart disease, left ventricular ejection fraction < 50%, secondary hypertension, coronary artery disease, cardiomyopathy, left or right bundle branch block, pacemaker rhythm, use of antiarrhythmic drugs other than beta‐blockers, unavailable electrocardiographic recordings, or electrocardiograms unsuitable for PTFV1 measurement.

The study protocol was approved by the Ethics Committee of İzmir Demokrasi University Buca Seyfi Demirsoy Training and Research Hospital (approval date: 28.01.2026; approval number: 2026/01‐08). The requirement for written informed consent was waived owing to the retrospective design of the study.

### 2.2. Clinical and Echocardiographic Data

Demographic, clinical, electrocardiographic, and echocardiographic data were obtained from hospital records. Age, sex, body mass index, heart rate, blood pressure measurements, and antihypertensive medication use were recorded. Mean arterial pressure was calculated using the following formula: mean arterial pressure = diastolic blood pressure + 1/3 × (systolic blood pressure − diastolic blood pressure).

Transthoracic echocardiographic parameters were retrieved from medical records. Left atrial diameter, right ventricular diameter, left ventricular ejection fraction, maximum aortic valve velocity, left ventricular mass index (LVMI), and mitral inflow parameters were recorded. The early‐to‐late mitral inflow velocity ratio was expressed as the E/A ratio. Left ventricular mass was calculated using the linear method recommended by the American Society of Echocardiography (ASE): LV mass (g) = 0.8 × {1.04 × [(IVSd + LVIDd + PWTd)^3^ − LVIDd^3^]} + 0.6. Left ventricular mass was indexed to body surface area to obtain the LVMI (g/m^2^). LVH was defined as an LVMI > 115 g/m^2^ in men and > 95 g/m^2^ in women, according to the recommendations of the ASE and the European Association of Cardiovascular Imaging (ASE/EACVI) [16].

### 2.3. Electrocardiographic Assessment and PTFV1 Measurement

Standard 12‐lead electrocardiograms were recorded at a paper speed of 25 mm/s and a calibration of 10 mm/mV. ECG recordings were reviewed in a digital environment and magnified to facilitate measurement. PTFV1 measurements were performed manually on the magnified digital ECG recordings in lead V1. PTFV1 was measured when the P‐wave was biphasic or had a clearly identifiable terminal negative component, using the TP segment as the isoelectric baseline. PTFV1 was calculated as the product of the duration and negative amplitude of the terminal negative P‐wave component: PTFV1 = terminal negative P‐wave duration × terminal negative P‐wave amplitude. The duration was measured in seconds and the amplitude in millimeters; therefore, PTFV1 was expressed as mm· s Figure 2 illustrates the measurement of the terminal negative P‐wave amplitude and duration used for PTFV1 calculation.

**FIGURE 2:**
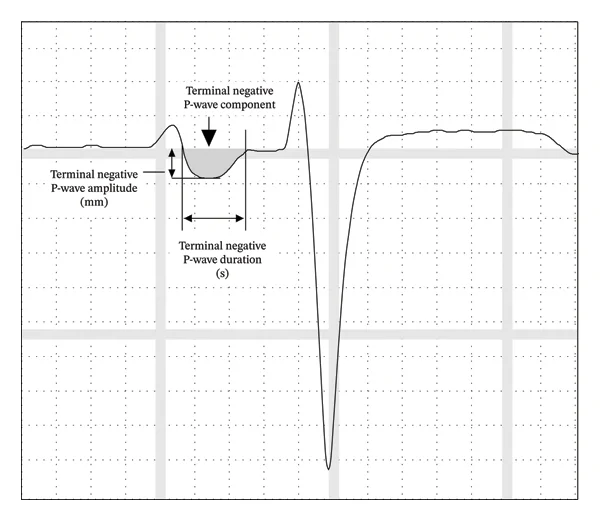
Schematic illustration of P‐wave terminal force in lead V1 (PTFV1) measurement. PTFV1 was calculated as the product of the duration of the terminal negative P‐wave component and its negative amplitude, using the TP segment as the isoelectric baseline (PTFV1 = terminal negative P‐wave duration × terminal negative P‐wave amplitude; unit: mm.s).

PTFV1 measurements used in the main analyses were performed by an experienced cardiologist blinded to clinical data and beta‐blocker treatment status. To assess interobserver reproducibility, a randomly selected subset of 50 ECGs was independently remeasured by a second experienced cardiologist who was also blinded to clinical data, treatment status, and the first observer’s measurements. Interobserver agreement was evaluated using the single‐measure intraclass correlation coefficient with a two‐way random‐effects model for absolute agreement. Bland–Altman analysis was performed to assess the mean interobserver difference and 95% limits of agreement between the two observers.

### 2.4. Definition of Abnormal PTFV1

In the primary analysis, PTFV1 was evaluated as a continuous variable. In a secondary analysis, abnormal PTFV1 was defined as PTFV1 ≥ 0.04 mm s, based on commonly used thresholds in previous studies evaluating PTFV1 as a marker of atrial abnormality and atrial fibrillation risk. Patients were then categorized as having normal PTFV1 values (< 0.04 mm.s) or abnormal PTFV1 values (≥ 0.04 mm.s) [11, 17, 18].

### 2.5. Statistical Analysis

Statistical analyses were performed using SPSS Version 27.0 (IBM Corp., Armonk, NY, USA). The distribution of continuous variables was assessed using visual methods and normality tests. Continuous variables were presented as mean ± standard deviation or median with interquartile range, according to data distribution. Categorical variables were expressed as numbers and percentages.

Comparisons between beta‐blocker users and nonusers were performed using the independent‐samples *t*‐test or Mann–Whitney *U* test for continuous variables, as appropriate. Categorical variables were compared using the chi‐square test or Fisher’s exact test when required. A two‐sided *p* value < 0.05 was considered statistically significant.

Multivariable linear regression analysis was performed to determine whether beta‐blocker use was independently associated with PTFV1. Age, sex, mean arterial pressure, LVMI, left ventricular ejection fraction, E/A ratio, and left atrial diameter were included as covariates. Regression results were reported as unstandardized coefficients, standard errors, standardized beta coefficients, 95% confidence intervals, and *p* values.

Because PTFV1 values may show a skewed distribution, a sensitivity analysis was performed using log‐transformed PTFV1 values. An additional heart‐rate‐adjusted model was also constructed to evaluate whether the association between beta‐blocker use and lnPTFV1 was independent of heart rate.

To account for potential treatment‐selection bias, a propensity score for beta‐blocker use was calculated using multivariable logistic regression. Age, sex, body mass index, mean arterial pressure, LVMI, left ventricular ejection fraction, E/A ratio, and left atrial diameter were included in the propensity score model. The association between beta‐blocker use and lnPTFV1 was then reassessed using a propensity score‐adjusted linear regression model.

For the secondary analysis, multivariable logistic regression was performed to evaluate the association between beta‐blocker use and abnormal PTFV1, defined as PTFV1 ≥ 0.04 mm s. Age, sex, mean arterial pressure, left ventricular ejection fraction, LVMI, E/A ratio, and left atrial diameter were included as covariates. Logistic regression results were reported as odds ratios with 95% confidence intervals and *p* values. Model fit was evaluated using the Hosmer–Lemeshow goodness‐of‐fit test.

## 3. Results

A total of 182 patients were included in the study, including 82 patients receiving β1‐selective beta‐blocker therapy and 100 patients not receiving beta‐blockers. Interobserver reproducibility was assessed in a randomly selected subset of 50 ECGs. Agreement between the two observers was excellent, with a single‐measure intraclass correlation coefficient of 0.969 (95% CI: 0.945–0.982, *p* < 0.001). Bland–Altman analysis demonstrated minimal mean interobserver bias (mean difference: −0.0003 mm.s), with 95% limits of agreement ranging from −0.0107 to 0.0101 mm.s.

No statistically significant between‐group differences were observed in age, sex, BMI, diabetes mellitus, concomitant ACE inhibitor/ARB, calcium‐channel blocker and diuretic use, renal function, left atrial and right ventricular dimensions, AVmax, LVMI, heart rate, E/A ratio, or systolic blood pressure (all *p* > 0.05). Serum creatinine (0.855 [0.770–0.983] vs. 0.860 [0.760–0.980] mg/dL, *p* = 0.956) and eGFR (82.45 ± 23.24 vs. 85.71 ± 22.05 mL/min/1.73 m^2^, *p* = 0.335) were similar between groups. Systolic blood pressure was also comparable (130 [128–140] vs. 132 [128.5–140] mmHg, *p* = 0.204), whereas diastolic blood pressure was modestly lower in beta‐blocker users (78 [75–82] vs. 80 [75–85] mmHg, *p* = 0.024).

PTFV1 values were significantly lower in patients receiving beta‐blocker therapy compared with those not receiving beta‐blockers (0.024 [0.015–0.036] vs. 0.030 [0.020–0.048] mm s, *p* = 0.005). Mean arterial pressure was also modestly lower in the beta‐blocker group (95.7 [92.0–100.0] vs. 97.2 [93.3–103.3] mmHg, *p* = 0.048). Although LVEF differed statistically between groups (65 [60–65] vs. 65 [63–65]%, *p* = 0.016), the absolute difference was small (Table 1). Among beta‐blocker users, PTFV1 values were similar between patients receiving metoprolol or bisoprolol and those receiving nebivolol (0.020 [0.010–0.025] vs. 0.024 [0.012–0.035] mm s, *p* = 0.287).

**TABLE 1:** Comparison of clinical, laboratory, electrocardiographic, and echocardiographic characteristics according to beta‐blocker use.

Variable	BB users (*n* = 82)	Non‐BB users (*n* = 100)	*p* value
Age, years	60.1 ± 10.0	59.1 ± 11.0	0.542
Female sex, *n* (%)	45 (54.9)	52 (52.0)	0.699
BMI, kg/m^2^	28.7 (27.0–30.9)	28.7 (26.7–31.3)	0.937
Diabetes mellitus, *n* (%)	8 (9.8)	11 (11.0)	0.785
ACEI/ARB use, *n* (%)	79 (96.3)	94 (94.0)	0.517^†^
CCB use, *n* (%)	55 (67.1)	64 (64.0)	0.665
Diuretic use, *n* (%)	42 (51.2)	56 (56.0)	0.520
Creatinine, mg/dL	0.855 (0.770–0.983)	0.860 (0.760–0.980)	0.956
eGFR, mL/min/1.73 m^2^	82.45 ± 23.24	85.71 ± 22.05	0.335
Systolic blood pressure, mmHg	130 (128–140)	132 (128.5–140)	0.204
Diastolic blood pressure, mmHg	78 (75–82)	80 (75–85)	**0.024**
MAP, mmHg	95.7 (92.0–100.0)	97.2 (93.3–103.3)	**0.048**
Heart rate, bpm	77.3 ± 7.9	79.3 ± 9.8	0.129
LA diameter, mm	36 (34–38)	35 (33–37)	0.206
RV diameter, mm	29 (27–31)	28 (25.5–30)	0.091
LVEF, %	65 (60–65)	65 (63–65)	**0.016**
AVmax, m/s	1.10 (1.10–1.20)	1.10 (1.10–1.20)	0.864
LVMI, g/m^2^	115.8 (103.6–118.5)	115.1 (105.5–119.0)	0.846
E/A ratio	0.83 (0.72–1.11)	0.78 (0.70–0.94)	0.072
PTFV1, mm⋅s	0.024 (0.015–0.036)	0.030 (0.020–0.048)	**0.005**

*Note:* Data are presented as mean ± standard deviation, median (Q1–Q3), or *n* (%), as appropriate. Continuous variables were compared using the independent‐samples *t*‐test or Mann–Whitney *U* test, as appropriate. Categorical variables were compared using the chi‐square test or Fisher’s exact test, as appropriate. AVmax, maximum aortic valve velocity; PTFV1, P‐wave terminal force in lead V1. Bold values indicate statistical significance (*p* < 0.05).

Abbreviations: ACEI = angiotensin‐converting enzyme inhibitor, ARB = angiotensin receptor blocker, BB = beta‐blocker, BMI = body mass index, CCB = calcium‐channel blocker, eGFR = estimated glomerular filtration rate, LA = left atrial, LVEF = left ventricular ejection fraction, LVMI, left ventricular mass index, MAP = mean arterial pressure, RV = right ventricular.

^†^Fisher’s exact test. Two‐sided *p* < 0.05 was considered statistically significant.

Multivariable linear regression analysis was performed to evaluate the association between beta‐blocker use and PTFV1 after adjustment for age, sex, mean arterial pressure, LVMI, left ventricular ejection fraction, E/A ratio, and left atrial diameter. Beta‐blocker use was associated with lower PTFV1 values in the adjusted model (*B* = −0.008, standardized *β* = −0.202, 95% CI −0.014 to −0.002, *p* = 0.009). However, the overall explanatory capacity of the model was limited (*R* ^2^ = 0.064; adjusted *R* ^2^ = 0.021), and the overall regression model was not statistically significant (*p* = 0.168). None of the other included covariates showed statistically significant individual associations with PTFV1 (Table 2). Accordingly, the beta‐blocker coefficient should be interpreted in the context of the limited explanatory performance of the overall model.

**TABLE 2:** Multivariable linear regression analysis of PTFV1.

Variable	*B*	Standard error	Standardized *β*	95% CI for *B*	*p* value
BB use	−0.008	0.003	−0.202	−0.014 to −0.002	**0.009**
Age (years)	−0.00003	0.000	−0.015	−0.000 to 0.000	0.864
Sex (female)	0.003	0.003	0.068	−0.004 to 0.009	0.409
LVMI (g/m^2^)	−0.00009	0.000	−0.064	−0.000 to 0.000	0.441
LVEF (%)	−0.0004	0.001	−0.065	−0.002 to 0.001	0.430
E/A ratio	0.0002	0.006	0.002	−0.012 to 0.012	0.977
MAP (mmHg)	0.0003	0.000	0.108	−0.000 to 0.001	0.159
LA diameter (mm)	0.0003	0.001	0.036	−0.001 to 0.001	0.656

*Note:* Model statistics: *R* ^2^ = 0.064; Adjusted *R* ^2^ = 0.021; *F* = 1.479; *p* = 0.168; Durbin–Watson = 1.623. E/A, early‐to‐late mitral inflow velocity ratio; PTFV1, P‐wave terminal force in lead V1.

Abbreviations: BB = beta‐blocker, CI = confidence interval, LA = left atrial, LVEF = left ventricular ejection fraction, LVMI = left ventricular mass index, MAP = mean arterial pressure.

A sensitivity analysis was performed using log‐transformed PTFV1 values. Beta‐blocker use remained associated with lower lnPTFV1 values (*B* = −0.267, standardized *β* = −0.206, 95% CI −0.462 to −0.072, *p* = 0.008). However, the overall regression model was not statistically significant (*p* = 0.208), indicating that this finding should be interpreted cautiously and should not be considered confirmation of a strongly explanatory multivariable model (Table 3). In an additional heart‐rate‐adjusted model, beta‐blocker use remained significantly associated with lower lnPTFV1 values (standardized *β* = −0.196, *p* = 0.009), whereas heart rate was not significantly associated with lnPTFV1 (standardized *β* = 0.076, *p* = 0.310). This analysis suggests that heart rate alone is unlikely to fully account for the observed association, although the limited explanatory performance of the models should be considered.

**TABLE 3:** Sensitivity analysis of beta‐blocker use and log‐transformed PTFV1.

Variable	*B*	Standard error	Standardized *β*	95% CI for *B*	*p* value
BB use	−0.267	0.099	−0.206	−0.462 to −0.072	**0.008**
Age (years)	0.000	0.005	−0.004	−0.011 to 0.010	0.960
Sex (female)	0.101	0.106	0.079	−0.109 to 0.311	0.342
LVMI (g/m^2^)	−0.002	0.004	−0.043	−0.011 to 0.006	0.604
LVEF (%)	−0.001	0.020	−0.005	−0.040 to 0.038	0.948
E/A ratio	−0.021	0.198	−0.008	−0.412 to 0.370	0.917
MAP (mmHg)	0.006	0.006	0.077	−0.006 to 0.017	0.311
LA diameter (mm)	0.005	0.019	0.021	−0.033 to 0.043	0.791

*Note:* Model statistics: *R* ^2^ = 0.060; Adjusted *R* ^2^ = 0.017; *F* = 1.381; *p* = 0.208; Durbin–Watson = 1.643. E/A, early‐to‐late mitral inflow velocity ratio; PTFV1, P‐wave terminal force in lead V1. Bold values indicate statistical significance (*p* < 0.05).

Abbreviations: CI = confidence interval, LA = left atrial, LVEF = left ventricular ejection fraction, LVMI = left ventricular mass index, MAP = mean arterial pressure.

In a further extended sensitivity analysis, the ln‐transformed PTFV1 model was additionally adjusted for diabetes mellitus, concomitant ACE inhibitor/ARB, calcium‐channel blocker and diuretic use, and eGFR, in addition to the covariates included in the primary model. Beta‐blocker group status remained associated with lnPTFV1 (*B* = 0.386, standardized *β* = 0.294, 95% CI 0.194–0.579, *p* < 0.001), with the positive coefficient reflecting higher lnPTFV1 values in non–beta‐blocker users according to the coding of the treatment variable. The extended model explained 14.7% of the variance in lnPTFV1 (*R* ^2^ = 0.147; adjusted *R* ^2^ = 0.081) and was statistically significant overall (*F*(13,168) = 2.219, *p* = 0.011). None of the additional clinical or treatment‐related covariates showed a statistically significant individual association with lnPTFV1. No problematic multicollinearity was observed (all VIFs < 1.5).

In the propensity score‐adjusted linear regression model, beta‐blocker use was also associated with lower lnPTFV1 values (*B* = −0.268, standardized *β* = −0.207, 95% CI −0.462 to −0.074, *p* = 0.007). This finding was directionally consistent with the primary analysis; however, it does not eliminate the possibility of residual confounding inherent to the observational study design.

### 3.1. Secondary Analysis of Abnormal PTFV1

As a secondary analysis, abnormal PTFV1 was defined as ≥ 0.04 mm s according to established literature thresholds. Among patients with normal PTFV1 values (< 0.04 mm⋅s), 57.5% (69/120) were receiving beta‐blocker therapy, whereas only 21.0% (13/62) of patients with abnormal PTFV1 values were receiving beta‐blockers (*X* ^2^ = 14.606, *p* < 0.001).

To further evaluate this association, a multivariable logistic regression analysis was performed including age, sex, mean arterial pressure, left ventricular ejection fraction, LVMI, E/A ratio, and left atrial diameter as covariates. After adjustment for the prespecified covariates, absence of beta‐blocker therapy was associated with higher odds of abnormal PTFV1 (OR 2.35, 95% CI 1.19–4.62, *p* = 0.013). None of the other covariates were significantly associated with abnormal PTFV1. This secondary analysis was consistent with the direction of the continuous PTFV1 analysis; however, given the observational design and modest sample size, it should be regarded as supportive rather than confirmatory (Table 4).

**TABLE 4:** Multivariable logistic regression analysis for abnormal PTFV1 (≥ 0.04 mm · s).

Variable	OR	95% CI	*p*
BB use	0.43^∗^	0.22–0.84	0.013
Age	1.01	0.97–1.05	0.611
Female sex	0.53	0.26–1.09	0.084
MAP	1.02	0.98–1.06	0.305
LVEF	0.98	0.85–1.11	0.710
LVMI	0.99	0.96–1.02	0.432
E/A ratio	1.32	0.36–4.90	0.675
LA diameter	1.04	0.91–1.18	0.554

*Note:* Model statistics: Nagelkerke *R* ^2^ = 0.089; Hosmer–Lemeshow goodness‐of‐fit test *p* = 0.052. E/A ratio, early‐to‐late diastolic transmitral flow velocity ratio.

Abbreviations: BB = beta‐blocker, CI = confidence interval, LA = left atrial, LVEF = left ventricular ejection fraction, LVMI = left ventricular mass index, MAP = mean arterial pressure, OR = odds ratio.

^∗^OR for beta‐blocker use was inverted from the original model coding for ease of clinical interpretation.

## 4. Discussion

The main findings of the present study can be summarized as follows. First, hypertensive patients with echocardiographically confirmed LVH who were receiving β1‐selective beta‐blocker therapy had lower PTFV1 values compared with patients not receiving beta‐blockers. Second, this association remained significant after adjustment for age, sex, mean arterial pressure, LVMI, left ventricular ejection fraction, E/A ratio, and left atrial diameter. Third, a similar direction of association was observed in analyses using log‐transformed PTFV1 values, after additional adjustment for heart rate, and in a propensity score‐adjusted model. Finally, β1‐selective beta‐blocker use was associated with a lower likelihood of abnormal PTFV1, defined as PTFV1 ≥ 0.04 mm s. Taken together, these findings suggest that β1‐selective beta‐blocker therapy is associated with a lower burden of atrial electrical abnormality as reflected by PTFV1 in hypertensive patients with LVH. The association between beta‐blocker use and lnPTFV1 was also observed after additional adjustment for diabetes mellitus, renal function, and concomitant antihypertensive therapies. However, the modest explanatory power of the model suggests that PTFV1 is likely influenced by additional clinical and biological factors beyond those included in the present analysis.

PTFV1 is an easily obtainable electrocardiographic marker that reflects the duration and amplitude of the terminal negative component of the P‐wave in lead V1. Although traditionally considered a marker of left atrial abnormality, recent evidence suggests that abnormal PTFV1 may reflect atrial electrical dysfunction rather than structural remodeling alone [18]. Previous studies have associated higher PTFV1 values with NOAF [19]. However, the present study did not assess incident atrial fibrillation, left atrial volume, or atrial strain. Although left atrial diameter and LVMI were not significantly associated with PTFV1 in the fitted model, these findings do not establish whether PTFV1 reflects structural or functional atrial changes in this cohort.

Previous research has linked hypertension and LVH to left atrial changes through mechanisms that may include increased afterload, impaired ventricular relaxation, elevated filling pressures, and altered atrial‐ventricular coupling [7–9]. Such mechanisms may contribute to atrial conduction abnormalities and the development of atrial fibrillation. In this context, PTFV1 provides an electrocardiographic measure of atrial electrical abnormality, but the present data cannot establish the underlying structural or hemodynamic changes. Comprehensive diastolic function and left atrial volume were not assessed; therefore, we cannot determine whether differences in ventricular filling properties or atrial structure contributed to the lower PTFV1 values observed among β1‐selective beta‐blocker users. The clinical relevance of this finding requires prospective evaluation with detailed echocardiographic assessment and atrial fibrillation outcomes.

The relationship between antihypertensive therapy and atrial fibrillation prevention remains complex. In previous research, blood pressure reduction and changes in ventricular structure or filling conditions have been proposed as pathways through which antihypertensive treatment might influence atrial fibrillation risk; these pathways were not evaluated in the present study. However, the effects of different antihypertensive drug classes on atrial fibrillation prevention have not been uniform across studies. Emdin et al. reported in a large meta‐analysis that antihypertensive therapy was associated with a reduced risk of atrial fibrillation, although the relative benefit of specific drug classes varied according to the clinical population studied [20]. In the LIFE study, angiotensin II receptor blockade reduced NOAF compared with atenolol in hypertensive patients with LVH [21]. These findings indicate that the antiarrhythmic implications of antihypertensive therapy may depend on patient phenotype, background cardiovascular risk, and the specific agents being compared. The present study does not directly compare beta‐blockers with a specific alternative antihypertensive class; rather, it evaluates the association between β1‐selective beta‐blocker use and PTFV1 in a selected hypertensive LVH population. In the present cohort, concomitant ACE inhibitor/ARB, calcium‐channel blocker, and diuretic use did not differ significantly between beta‐blocker users and nonusers. Although this reduces concern regarding major imbalances in background antihypertensive therapy, residual confounding related to treatment selection and unmeasured differences in treatment intensity cannot be excluded.

Diastolic blood pressure and mean arterial pressure were modestly lower in beta‐blocker users, whereas systolic blood pressure did not differ significantly between groups. Although mean arterial pressure was included in the multivariable models, the possibility that differences in hemodynamic load contributed in part to the observed PTFV1 differences cannot be completely excluded.

Several mechanisms may explain the association between *β*1‐selective beta‐blocker use and lower PTFV1. Increased sympathetic activation may promote atrial electrical instability, shorten atrial refractory periods, increase conduction heterogeneity, and facilitate atrial arrhythmogenesis. β1‐Selective beta‐blockers may attenuate these processes by reducing sympathetic stimulation of the myocardium, stabilizing atrial electrophysiology, and lowering myocardial oxygen demand. Experimental data have shown that excessive β1‐adrenergic receptor stimulation may contribute to cardiomyocyte hypertrophy and maladaptive remodeling [22]. Therefore, selective β1‐receptor blockade may theoretically reduce the electrophysiological consequences of adrenergic overstimulation in hypertensive LVH.

The β1‐selective beta‐blocker group included patients receiving metoprolol, bisoprolol, or nebivolol. These agents differ in their pharmacological properties. For example, lipophilicity may affect tissue penetration [23], whereas nebivolol has nitric oxide‐mediated vasodilatory properties and has shown vascular and antifibrotic effects in experimental studies [24, 25]. A previous meta‐analysis has also examined the effects of beta‐blockers on left ventricular mass in hypertension [26]. However, the present study did not compare PTFV1 across individual agents or assess changes in left ventricular mass over time. Information on beta‐blocker dose and treatment duration beyond the minimum 3‐month criterion was unavailable. Therefore, these potential mechanisms cannot be linked to the observed difference in PTFV1, and we cannot determine whether the association varied among metoprolol, bisoprolol, and nebivolol. In an exploratory subgroup analysis, PTFV1 did not differ significantly between nebivolol users and those receiving metoprolol or bisoprolol. However, the subgroup sample sizes were limited, and agent‐specific or dose‐related effects cannot be excluded.

The persistence of the association after heart‐rate adjustment is noteworthy. Beta‐blockers are expected to reduce heart rate, and heart rate reduction could theoretically influence P‐wave indices by altering atrial conduction dynamics. However, in the present study, heart rate was not significantly associated with lnPTFV1 in the heart‐rate‐adjusted model, whereas beta‐blocker use remained independently associated with lower lnPTFV1. This suggests that the relationship between β1‐selective beta‐blocker therapy and PTFV1 may not be solely attributable to heart rate reduction. Nevertheless, because heart rate was assessed from resting clinical data and the study was retrospective, more detailed rhythm monitoring data would be required to fully clarify this mechanism.

The secondary analysis using abnormal PTFV1 as a categorical outcome further supports the main findings. Patients with abnormal PTFV1 were less frequently receiving beta‐blocker therapy, and beta‐blocker use was associated with lower odds of abnormal PTFV1 after adjustment for relevant clinical and echocardiographic covariates. This result is consistent with the continuous‐variable analysis and suggests that β1‐selective beta‐blocker therapy may be associated not only with lower absolute PTFV1 values but also with a lower probability of crossing a clinically recognized abnormal threshold. However, abnormal PTFV1 should not be interpreted as a definitive diagnostic marker of atrial remodeling or future atrial fibrillation in an individual patient. Rather, it may provide complementary information when interpreted together with clinical characteristics, echocardiographic findings, and overall cardiovascular risk.

The findings of the present study are also consistent with previous observations showing favorable effects of beta‐blocker therapy on electrocardiographic indices of atrial conduction. Camsari et al. reported that long‐term metoprolol therapy was associated with reductions in maximum P‐wave duration and P‐wave dispersion in patients with congestive heart failure [15]. Since P‐wave duration is one of the components contributing to PTFV1, improvement in atrial conduction indices may represent one possible pathway linking beta‐blocker therapy with lower PTFV1. More recently, Koraćević et al. emphasized the potential role of beta‐blockers in hypertensive LVH and atrial fibrillation prevention, particularly through mechanisms extending beyond anatomical LVH regression [27]. The present findings add to this concept by suggesting that β1‐selective beta‐blocker use may be associated with a more favorable atrial electrical profile as reflected by PTFV1.

Several limitations should be acknowledged. First, this was a retrospective, observational, single‐center study; therefore, causality cannot be established. The decision to prescribe β1‐selective beta‐blockers was not randomized and may have been influenced by clinical characteristics that were not fully captured in the available data. The specific clinical indication for beta‐blocker initiation could not be reliably determined for all patients from the retrospective records; therefore, indication‐related treatment‐selection bias cannot be excluded. The exact duration of hypertension could not be reliably determined from the retrospective records; therefore, the potential influence of cumulative long‐term blood pressure exposure could not be fully assessed. Although multivariable adjustment and propensity score adjustment were performed, residual confounding and treatment‐selection bias cannot be excluded. Second, the sample size was relatively modest, particularly for categorical analysis of abnormal PTFV1. Third, although beta‐blocker use was significantly associated with PTFV1, the overall explanatory power of the regression models was limited, indicating that PTFV1 is influenced by additional factors not included in the present analysis. Fourth, detailed information regarding beta‐blocker dose, treatment adherence, and treatment duration beyond the minimum 3‐month criterion was not available. In addition, the effects of individual β1‐selective beta‐blockers were not analyzed separately; therefore, potential agent‐specific or dose‐related differences among metoprolol, bisoprolol, and nebivolol could not be determined. Fifth, a comprehensive assessment of left ventricular diastolic function was not available. Because the E/A ratio alone is insufficient to diagnose or grade diastolic dysfunction, we could not determine whether ventricular filling properties contributed to the observed association with PTFV1. Left atrial volume index, left atrial strain, cardiac magnetic resonance imaging, and biomarkers of fibrosis or neurohormonal activation were also unavailable; therefore, we could not evaluate atrial structural or functional remodeling directly. Finally, the study did not include longitudinal follow‐up for NOAF or other clinical outcomes. Prospective studies are needed to determine whether lower PTFV1 among β1‐selective beta‐blocker users translates into reduced atrial fibrillation risk.

In summary, β1‐selective beta‐blocker use was independently associated with lower PTFV1 values and a lower likelihood of abnormal PTFV1 in hypertensive patients with LVH. These findings suggest a potential favorable association between β1‐selective beta‐blocker therapy and atrial electrical remodeling. However, given the observational design and modest explanatory power of the models, the results should be interpreted as hypothesis‐generating rather than definitive evidence of a protective effect.

## 5. Conclusion

In this retrospective observational study of hypertensive patients with echocardiographically confirmed LVH, β1‐selective beta‐blocker use was associated with lower PTFV1 values and lower odds of abnormal PTFV1 (≥ 0.04 mm s). The association was observed after adjustment for measured clinical and echocardiographic variables and in analyses using log‐transformed PTFV1, additional heart‐rate adjustment, and propensity score adjustment. These consistent findings support further investigation of the relationship between β1‐selective beta‐blocker use and atrial electrical markers in hypertensive patients with LVH. Given the observational design and limited explanatory capacity of the models, they should not be interpreted as evidence of improved atrial remodeling or reduced atrial fibrillation risk. Prospective studies incorporating comprehensive atrial assessment and longitudinal outcomes are needed.

## Author Contributions

Conceptualization: Sedat Taş, Erkan Alpaslan, Onur Dalgıç, Lütfullah Candan, Ahmet Rıdvan Özdemir. Methodology: Erkan Alpaslan, Ümmü Taş, Sedat Taş, Onur Dalgıç, Ahmet Rıdvan Özdemir. Formal analysis: Onur Dalgıç, Ömer Özkan Duman, Sedat Taş, Ümmü Taş, Mehmet Eyüboğlu Investigation: Erkan Alpaslan, Mehmet Eyüboğlu. Data curation: Sedat Taş, Ümmü Taş, Ömer Özkan Duman, Onur Dalgıç, Lütfullah Candan, Ahmet Rıdvan Özdemir. Resources: Sedat Taş, Ömer Özkan Duman, Lütfullah Candan Visualization: Sedat Taş, Mehmet Eyüboğlu, Lütfullah Candan. Validation: Ahmet Rıdvan Özdemir. Project administration: Mehmet Eyüboğlu, Erkan Alpaslan, Ümmü Taş. Supervision: Ümmü Taş, Mehmet Eyüboğlu. Writing – original draft: Ümmü Taş, Sedat Taş, Erkan Alpaslan, Mehmet Eyüboğlu, Ömer Özkan Duman, Onur Dalgıç, Lütfullah Candan, Ahmet Rıdvan Özdemir. Writing – review & editing: Sedat Taş, Erkan Alpaslan.

## Funding

This research received no specific grant from any funding agency in the public, commercial, or not‐for‐profit sectors.

## Disclosure

All authors reviewed and approved the final version of the manuscript.

## Ethics Statement

This study was conducted in accordance with the principles of the Declaration of Helsinki and was approved by the relevant institutional ethics committee. The ethics committee approval details are provided in the Methods section.

## Consent

The requirement for informed consent was waived due to the retrospective design of the study and the use of anonymized patient data.

## Conflicts of Interest

The authors declare no conflicts of interest.

## Data Availability

The data that support the findings of this study are available from the corresponding author upon reasonable request.
